# Microsatellites Cross-Species Amplification across Some African Cichlids

**DOI:** 10.1155/2012/870935

**Published:** 2012-06-04

**Authors:** Etienne Bezault, Xavier Rognon, Karim Gharbi, Jean-Francois Baroiller, Bernard Chevassus

**Affiliations:** ^1^UMR 110, Cirad-Ifremer INTREPID, 34398 Montpellier, France; ^2^INRA, UMR 1313 Génétique Animale et Biologie Intégrative, 78352 Jouy-en-Josas, France; ^3^Department of Biology, Reed College, Portland, OR 97202, USA; ^4^AgroParisTech, UMR 1313, Génétique Animale et Biologie Intégrative, 75231 Paris, France; ^5^Institute of Evolutionary Biology, School of Biological Sciences, University of Edinburgh, Edinburgh EH9 3JT, UK

## Abstract

The transfer of the genomic resources developed in the Nile tilapia, *Oreochromis niloticus*, to other Tilapiines *sensu lato* and African cichlid would provide new possibilities to study this amazing group from genetics, ecology, evolution, aquaculture, and conservation point of view. We tested the cross-species amplification of 32 *O. niloticus* microsatellite markers in a panel of 15 species from 5 different African cichlid tribes: Oreochromines (*Oreochromis, Sarotherodon*), Boreotilapiines (*Tilapia*), Chromidotilapines, Hemichromines, and Haplochromines. Amplification was successfully observed for 29 markers (91%), with a frequency of polymorphic (P_95_) loci per species around 70%. The mean number of alleles per locus and species was 3.2 but varied from 3.7 within *Oreochromis* species to 1.6 within the nontilapia species. The high level of cross-species amplification and polymorphism of the microsatellite markers tested in this study provides powerful tools for a wide range of molecular genetic studies within tilapia species as well as for other African cichlids.

## 1. Introduction

African cichlid fish are of extreme interest for both evolutionary biology and applied genetics purposes, including amazing models for speciation, adaptation, behaviour and neurosciences [1–5] as well as groups of major importance for aquaculture and fisheries (strain selection and improvement, stock assessment, etc.) [6–10]. A wide range of structural and functional genomic resources have been developed for cichlids in the past 15 years, predominantly in the Nile tilapia, *Oreochromis niloticus* [11–14]. While genome sequencing projects are in progress for several African cichlids, the transfer of genomic resources from *O. niloticus* across the entire group of tilapias *sensu lato* as well as other African cichlid tribes would provide powerful tools to support a wide range of evolutionary biology studies, including comparative phylogenetics, genome mapping, evolution of gene family sequence and expression, candidate gene analyses for adaptation, and population genetics.

Microsatellite markers are one of the most interesting resources to transfer across lineages, as they can provide numerous locus-specific molecular markers and putatively homologous sequences across taxa. In addition to their high level of polymorphism, the evolutionary conservation of the flanking region of microsatellite loci allows large-scale heterospecific amplification [15, 16], as previously shown in various animal groups, particularly fish [17–19]. However, the rate of cross-species amplification varies widely among taxonomic groups and loci [18, 20]. In addition to their application in population genetics, conserved microsatellite markers are particularly useful for population, species or hybrid identification (especially at early developmental stages) and candidate-marker analysis, comparative genetic mapping, and QTL analysis. Furthermore, compared to anonymous multilocus genomic markers (RFLP, AFLP, ISSR) and SNPs, microsatellites present the important advantages of (i) being highly reproducible and very easily transferable between laboratory (with limited equipment and computational requirement), (ii) providing a high polymorphism information contain (PIC) per locus, and (iii) being highly cost efficient when only a small number of loci are needed. For these reasons, microsatellites markers are likely to remain popular for a wide range of ecology and evolutionary studies (e.g., relatedness and parentage analysis, population diversity and demography assessment, noninvasive genetic analysis, and conservation).

Since the first publication of microsatellite markers cloned in *O. niloticus* [13], thousands have been published and more than 500 have been positioned onto the genetic map of *O. niloticus* and the closely related *O. aureus* [14, 21]. These microsatellites have been used to map traits of interest, such as sex determination factors [22, 23], and have also been found to influence the expression of genes associated to physiological adaptation [24].

Outside the tilapias, microsatellite markers have been developed in a few different Haplochromines species: *Copadichromis cyclicos* [25], *Tropheus moorii* [19], *Pseudotropheus zebra* [26], *Astatoreochromis alluaudi* [27], *Pundamilia pundamilia* [28], *Metriaclima zebra* [29], *Pseudocrenilabrus multicolor* [30], *Paralabidochromis chilotes* [31], and *Astatotilapia burtoni* [32]. However these studies reported a smaller number of markers than that in Nile tilapia. The use of microsatellite markers in Haplochromines has been almost strictly restricted to descriptive population genetics and parentage/relatedness analysis, which represent only a subset of the possibilities offered by having a large set of genome-anchored microsatellite markers, as available for *O. niloticus*.

Additionally, microsatellites developed outside tilapias were derived exclusively from the most species-rich group of African cichlids and there are very limited genomic resources in all the other “under-studied” African cichlid tribes [33–35].

Considering the central position occupied by the Tilapiines *sensu lato* in the African cichlid phylogeny [38], their large diversity within at least 3 monophyletic clades [39–41], and the important number of species involved in population transfers, hybridisation, and/or invasion [8, 42], we decided to investigate the cross-species amplification efficiency of Nile tilapia microsatellites among the different groups of the Tilapiines *sensu lato* as well as three other African cichlid tribes, to extend the use/availability of this resource across a wide range of African cichlid species, including “under-studies” groups. The panel of species investigated then spans a large section of the African cichlid radiation, with an estimated overall divergence time of 33.4–63.7 Myrs [41, 43].

## 2. Material and Methods

Tests of cross-species amplification were conducted in a panel of 15 African cichlid species, representing all three major genera of Tilapiines *sensu lato*: 7 *Oreochromis*, 2 *Sarotherodon*, both genera belonging to the Oreochromines, and 3 *Tilapia *(*Coptodon*), belonging to the Boreotilapiines; as well as representatives of 3 other African cichlid tribes, including the derived Haplochromines, and two more basal tribes, the Chromidotilapiines and the Hemichromines (see details in Table 1). Analyses were conducted using 3 to 9 individuals per species (Table 1). Genomic DNA was extracted from fin clips stored in ethanol using a standard phenol-chloroform protocol [44].

The panel of 32 microsatellites was selected from the markers isolated in *O. niloticus* [13]. Genotyping was obtained by PCR amplification with radioactive (P^33^) labeled primers [44, 45]. Allele variants were separated on 6% acrylamide gel electrophoresis. For each marker, the annealing temperature and MgCl_2_ concentration were adjusted to optimise the efficiency of PCR amplification based on *O. niloticus * and two others species: one closely related among *Oreochromis* (*O. mossambicus*) and one distantly related among the Oreochromines (*S. melanotheron*). Cross-species amplifications were carried out using these conditions in the 15 studied species (Table 2). For each microsatellite marker, the amplification success has been estimated qualitatively on a 4-level scale based on the quality of the electrophoresis pattern across the test individuals (i.e., “++” for strong and sharp amplification pattern, “+” for good quality pattern with some stutters, echo-alleles or low intensity, “−” for high variance of amplification quality across individuals, very high level of stutter, and/or high frequency of null alleles, and “−−” very poor quality pattern, nonspecific or lack of, amplification). For each locus by species combination (*n* = 480), we assessed the amplification success and counted the number of different alleles among individuals. The presence of putative null alleles (i.e., nonamplified alleles) was inferred when a few individuals consistently showed an absence of allele amplification while other individuals from the same species showed high-quality amplification pattern or in the complete absence of heterozygous individuals. Echo-alleles (i.e., supplementary allele coamplifying across individuals producing amplification pattern consistently representing 2 or 4 alleles per individuals, with the longest allele separated from the shortest “cosegregating” allele by an identical length across individuals/alleles) were also identified. Furthermore, the rate of amplification success, the frequency of polymorphic loci (P_95_), and the mean number of allele per locus were calculated per species, genus, and tribe across all studied microsatellites markers.

## 3. Results and Discussion

Very high rates of microsatellite amplification and polymorphism were observed (both 97%), in the Nile tilapia, with a mean number of alleles per locus of 4.3. Across the 14 other test species, 29 loci gave good quality amplifications (91%-Tables 2 and 3), while 3 markers (9%) showed a high discrepancy of amplification efficiency and/or unclear amplification pattern (Table 2; *see details in supplementary material* which is available online at doi:10.1155/2012/870935: Table S1). Excluding the Nile tilapia, the average intraspecific rate of successful amplification and polymorphism across the panel of 32 markers was more than 70% (Table 3).

The expected relationship between the success of cross-species amplifications and evolutionary distance from marker cloning species [15, 20] was observed, reflecting the phylogenetic relationships between the different groups of African cichlids [39–41] (Table 3; *see details in supplementary material: *Table S2). Within the Tilapiines *sensu lato*, species from both mouth-brooder genera (i.e.,* Oreochromis* and *Sarotherodon*), constitutive of the monophyletic clade of the Oreochromines diverged 12.8–21.4 Myrs ago, showed very high and similar amplification (88% and 86%, resp.) and polymorphism (76% and 85%, resp.) rates, whereas species from the genus *Tilapia*, belonging to the Boreotilapiines with a divergence time from Oreochromines of 30.6–39.6 Myrs, showed lower rates of amplification (67%) and polymorphism (59%). The three other African cichlid tribes exhibited lower values for amplification and polymorphism rates: 38% and 50%, respectively, in the more derived lineage, Haplochromines, whereas a more heterogeneous pattern was found for the two more basal lineages, Chromidotilapiines (i.e., 47% and 20%, resp.) and Hemichromines (i.e., 19% and 50%, resp.). Allelic diversity varied with the same trends with a mean number of alleles per locus and species ranging from 3.7 and 3.3, respectively, for *Oreochromis *spp. and *Sarotherodon *spp. to 2.4 for *Tilapia *spp. and 1.6 in average (from 1.4 to 2.3) for the non-Tilapiines groups. The frequency of loci with putative null alleles also appeared to increase in the more distant species (*supplementary material: *Table S2). Rather than strictly reflecting reductions in polymorphism and/or the loss of the marker loci with increasing phylogenetic distance from the species in which the marker was cloned, these relationships are caused by mutations in the flanking regions complementary to the PCR primers. The conservation of microsatellites loci in the genomes has been shown to be potentially very long, and anyway much longer than the divergence time allowing successful cross-species amplification based on a given pair of primers, generally designed based on the only knowledge of the locus sequence in the species of cloning. The global success of cross-species amplification of a given microsatellite marker and/or the recovery of its different allelic variant (i.e., elimination of null allele) could then be enhanced in target species by either a specific optimisation of the amplification conditions or the modification of the sequence of the primers. This is especially appropriate when target species are distantly related to the cloning species of the markers and initial cross-species tests reveal low level of polymorphism with potentially high frequency of null allele (which would heavily bias any allele frequency-based estimates). 

**Figure 1:**
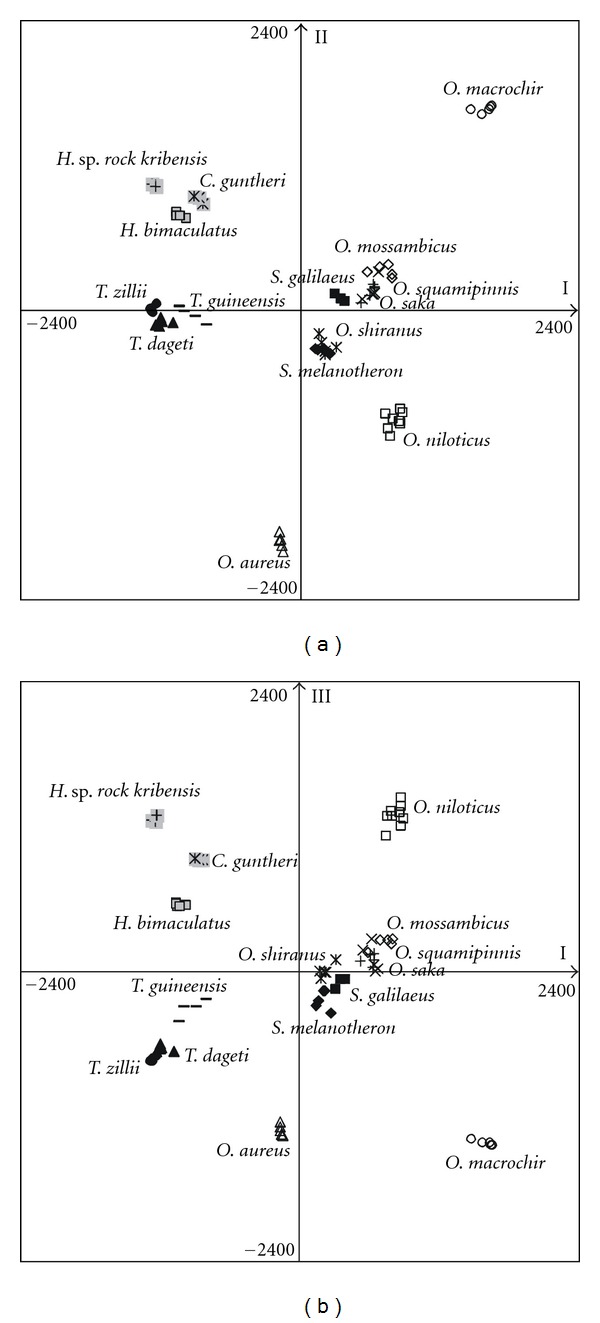
Clustering of the 15 study species based on multilocus diversity: correspondence analysis based on the individual genotypes over the 29 microsatellites loci successfully amplified and performed on the barycentre of the species: (a) factorial plane F1-F2 and (b) factorial planes F1–F3.

To represent the multi-locus pattern of genetic diversity across the 15 study species, we performed a population-based correspondence analysis using the software Genetix [46]. This multivariate analysis conducted on the genotype matrix allows to represent the clustering pattern among the different species groups, as well as among individuals within each of them in a factorial space (F1, F2, F3). This analysis allowed to clearly resolve the different species, except for *O. saka* and *O. squamipinnis* which are highly overlapping in the factorial space (Figure 1). Three separate groups of species were defined: the Oreochromines species, with all *Oreochromis* and *Sarotherodon* species, the Tilapia species, and all non-Tilapiines species. This clustering pattern reflects the phylogenetic relationships between the two tribes of Tilapiines *sensu lato*, that is, Oreochromines and Boreotilapiines. However the clustering of the three other tribes, which represent the most distant taxa from the source species, reveals the influence of the overall reduced polymorphism in highly distant taxa. This points out the limits of microsatellite size polymorphisms to estimate genetic divergence and/or phylogenetic relationship between too distantly related taxa, due to allele size homoplasy and/or increase of null allele frequency [19].

## 4. Conclusion

This study provides a quantitative estimate of the transferability of *O. niloticus*-derived microsatellites markers across 5 divergent African cichlid tribes, from the highly studied Haplochromines group to less studied tribes as Oreochromines, Boreotilapiines, Chromidotilapiines, and Hemichromines. The high rate of cross-species amplification and polymorphism highlights the usefulness of microsatellites markers for comparative genetic studies within Oreochromines and other African cichlids tribes, including stock/species identification, comparative genome mapping, candidate genes, or hybridisation surveys. Despite the fast growing opportunities to produce large-scale genomic data in nonmodel organisms, we believe that highly polymorphic, locus-specific markers such as microsatellites will continue to be useful for a wide range of genetic analyses in African cichlids.

## Supplementary Material

The supplementary material provides the details about the efficiency of amplification for every locus by species combination, including also the corresponding level of polymorphism and allele size (Table S1). Furhtermore, the results of cross–specific amplification for each tested locus are provided for genus and tribes level (Table S2). Finally the frequency of shared alleles across species at genus and/or tribes level is provided.

## Figures and Tables

**Table 1:** Species studied for cross-species amplification tests, with geographic origin, and number of samples analysed per species.

Lineages Genus Species		Geographic origin	*n*
Oreochromines			
* Oreochromis*			
*O. *(*Oreochromis*)* niloticus *		Bouake (Cote d'Ivoire)*	9
*O. *(*Oreochromis*)* aureus *		Lake Manzala (Egypt)	5
*O. *(*Oreochromis*)* mossambicus *		Mozambique	5
*O. *(*Oreochromis*)* shiranus *		Lake Malawi	5
*O. *(*Nyasalapia*)* macrochir *		Bouake (Cote d'Ivoire)**	5
*O. *(*Nyasalapia*)* saka *		Lake Malawi	5
*O. *(*Nyasalapia*)* squamipinnis *		Lake Malawi	5
* Sarotherodon*			
*S. *(*Sarotherodon*)* galilaeus *		Bamako (Niger)	3
*S. *(*Sarotherodon*)* melanotheron *		Ébrié Lagoon (Ivory Cost)	5
Boreotilapiines			
* Tilapia*			
*T. *(*Coptodon*)* dageti *		Bamako (Niger)	5
*T. *(*Coptodon*)* guineensis *		Ivory Cost/Senegal	4
*T. *(*Coptodon*)* zillii *		Lake Manzala (Egypt)	5
Haplochromines			
* Haplochromis*			
*Haplochromis sp. “rock kribensis” *		Lake Victoria	3
Chromidotilapines			
* Chromidotilapia*			
*Chromidotilapia guntheri *		Bamako (Niger)	3
Hemichromines			
* Hemichromis*			
*Hemichromis bimaculatus *		Bandama (Ivory Cost)	5

Introduced stocks: *with mixed origin (Volta and Nile) [36]; **from wild population (RDC) [37].

**Table 2:** Microsatellite loci tested for cross-species amplification with indications of repeat structure observed in *O. niloticus* (according to Lee and Kocher, [13]), allele size range of the amplified fragment across all tested species, PCR and electrophoresis conditions (labeled primer, annealing temperature/magnesium concentration (mM)/electrophoresis Volt-hour), and amplification quality obtained after PCR optimisation tests (from very good ++ to poor −−; see detail of the categories in main text); loci presenting a wide cross-species amplification efficiency are in bold.

Loci	GenBank access No.	Structure	Range (bp)	PCR and electrophoresis conditions	Amplification efficiency
**UNH-008**	G31346	Perfect	196–236	R* 56/1.2/6000	++
**UNH-102**	G12255	Perfect	132–185	R* 50/1.2/4500	++
**UNH-103**	G12256	Perfect	171–260	R* 48/1.2/6000	+
**UNH-106**	G12259	Compound	115–189	R* 50/1.2/3500	+
**UNH-115**	G12268	Compound	100–146	F* 50/1.5/3500	++
**UNH-117**	G12270	Interrupted	108–146	R* 5411.2/4500	++
UNH-120	G12273	Compound	—	R* 48/2/—	− −
**UNH-123**	G12276	Perfect	142–232	F* 48/1.2/4500	++
**UNH-124**	G12277	Perfect	295–324	F* 54/1.2/7500	++
**UNH-125**	G12278	Compound	134–198	R* 48/1.5/4500	+
**UNH-129**	G12282	Interrupted	180–253	R* 48/1.2/4500	+
**UNH-130**	G12283	Perfect	174–242	R* 50/1.2/4500	+
UNH-131	G12284	Perfect	283–303	F* 48/2/6000	−
**UNH-132**	G12285	Perfect	100–134	R* 52/1.2/3500	+
**UNH-135**	G12287	Interrupted	124–284	R* 50/1.5/4500	+
**UNH-138**	G12290	Perfect	144–250	R* 48/1.5/4500	+
**UNH-142**	G12294	Interrupted	142–192	F* 48/1.2/4500	++
**UNH-146**	G12298	Interrupted	111–149	F* 60/1/3500	++
**UNH-149**	G12301	Perfect	143–225	R* 48/1.5/4500	+
**UNH-154**	G12306	Perfect	98–176	R* 50/1.2/3500	++
**UNH-159**	G12311	Perfect	205–267	R* 55/1.2/6000	++
**UNH-162**	G12314	Perfect	125–252	R* 48/1.5/6000	++
**UNH-169**	G12321	Interrupted	124–240	R* 54/1.2/3500	++
**UNH-173**	G12325	Perfect	124–188	F* 55/1.2/4500	+
**UNH-174**	G12326	Perfect	146–187	F* 48/1.5/4500	++
**UNH-189**	G12341	Perfect	135–208	R* 52/1.2/4500	+
**UNH-190**	G12342	Compound	133–202	R* 60/1/4500	+
UNH-193	G12386	Perfect	—	R* 48/2/3500	− −
**UNH-197**	G12348	Interrupted	154–228	R* 50/1.2/4500	+
**UNH-207**	G12358	Interrupted	90–198	R* 60/1.2/3500	++
**UNH-211**	G12362	Perfect	82–194	R* 48/1.5/3500	++
**UNH-216**	G12367	Perfect	126–212	R* 52/1.2/3500	++

**Table 3:** Results of cross-species amplification performed over the 32 tested microsatellite loci on the 15 African cichlid species studied, including amplification rate, polymorphism rate, and mean number of alleles per locus, estimated per genus and tribe.

Groups	*N* species	Amplification rate	Polymorphism (P_95_)	Mean allele number per locus		% shared alleles per
				Per group	Per species	
*O. niloticus*		97%	97%	—	4.3	—

*Oreochromis *spp.*	6	88%	76%	17.8	3.7	37%
*Sarotherodon *spp.	*2*	86%	85%	6.4	3.3	9.2%
*Tilapia *spp.	*3*	67%	59%	6	2.4	19.7%
Tilapiines*	11	82%	74%	24.3	3.7	20.5%
non-Tilapiines	3	34%	36%	3.4	1.6	2.3%
Haplochromines	1	38%	50%	—	1.6	—
Chromidotilapines	1	47%	20%	—	1.4	—
Hemichromines	1	19%	50%	—	2.3	—

Total*		72%	70%	25.7	3.2	5.3%

*Excluding *O. niloticus.*
