# Supplementary material for: Microsatellites Cross-Species Amplification across Some African Cichlids
Source: Int J Evol Biol. 2012 Jun 4;2012:870935. doi: 10.1155/2012/870935 (PMC3373121; doi:10.1155/2012/870935)
Supplement: Supplementary file 1 — The supplementary material provides the details about the efficiency of amplification for every locus by species combination, including also the corresponding level of polymorphism and allele size (Table S1). Furhtermore, the results of cross–specific amplification for each tested locus are provided for genus and tribes level (Table S2). Finally the frequency of shared alleles across species at genus and/or tribes level is provided. [file 870935.f1.pdf]

SUPPLEMENTARY MATERIALS

Table S1 Results of cross-species amplification at species level; for each loci, the amplification quality and the number of alleles per species are given; and overall loci, the average rates of amplification and polymorphism (p>0.95), and the mean number of alleles per locus are provided - expected null alleles (N) and amplification of echo-allele (echo – i.e. supplementary longer length amplified allele always co-appearing with a given allelle) are also reported.

Species		*Oreochromis*							*Sarotherodon*		*Tilapia*			*Hemichromis*	*Chromidotilapia*	*Haplochromis*
Loci		*niloticus*	*aureus*	*mossambicus*	*shiranus*	*saka*	*squamipinnis*	*macrochir*	*melanotheron*	*galilaeus*	*zillii*	*dageti*	*guineensis*	*bimaculatus*	*guntheri*	*sp. rockkribensis*
**UNH-008**	quality	++	++	++	++	++	++	++	++	++	++	++	++	++	++	++
	# all.	3	1	1+N	5	6	6	2	2	6	1	1	1	2	1	1
	all.range	206-222	224	198	212-220	206-236	204-222	201-203	218-224	203-221	202	202	202	215-216	196	206
**UNH-102**	quality	++		++	++	++	++	++	++	-				--	++	++
	# all.	4	N	2+N	7	7+N	7	2	5	2+N	N	N	N	2+N	1	1
	all.range	147-175		163-167	147-177	151-185	151-177	159-171	151-167	159-161				157-161	141	132
**UNH-103**	quality	++	++	++	++	++	++	++	++	+						
	# all.	3	2	3	8	10	10	3	5	4+N	N	N	N	N	N	N
	all.range	206-220	198-222	182-224	206-252	180-240	184-260	182-234	171-240	178-186						
**UNH-106**	quality	++	++	++	++	++	++	++	++	++	++	++	+	--	++	++
	# all.	4	4	2+N	6	4	5	1	4+N	5+N	2	1	4+N	1+N	4	3
	all.range	131-141	155-189	115-117	155-171	127-147	119-149	119	125-135	125-143	125-173	123	137-163	133	137-147	157-163
**UNH-115**	quality	++	++	++	++	++	++	++	++	++	++	++	+	-	++	
	# all.	3	1	3	4	7	3	1	1	5+N	1	1	2	1	1	N
	all.range	128-146	122	112-140	122-131	112-132	120-127	118	124	114-128	128	128	124-128	100	120	
**UNH-117**	quality	++	++	++	++	++	++	++	++	++	++	++	++	-	++	-
	# all.	1	1	1	1+N	2	1	1	2	2	1	1	1	1	1	1
	all.range	120	120	120	120	118-120	120	120	120-124	116-122	112	116	116	128	108	146
UNH-120	quality	-	-	-	-	-	-	-	-	--	-	-	-	--	--	--
	# all.	5	3	2	8	11	8	2	2	2	1	1	1	N	N	2
	all.range															
**UNH-123**	quality	++	++	++	++	++	++	++	++	++		++	++	+	++	++
	# all.	6	4+N	3	3+N	4	6	2	2	5	N	2	1	1	1	1
	all.range	154-212	195-232	184-206	162-174	162-184	166-178	176-180	153-162	162-196		156-162	162	144	142	160
**UNH-124**	quality	++		++		+	+	+	++	++						
	# all.	4	N	3	N	1	2	1	1	5+N	2+N	N	N	N	N	N
	all.range	295-313		307-313		296	296-298	296	310	310-324	310-321					
**UNH-125**	quality	++	++		++			+	++		++	++	++			++
	# all.	7	1	N	3	N	N	2	4	2+N	1	3	2	N	N	1
	all.range	134-170	158		138-142			174-198	142-166	156-158	154	158-162	154-186			194
**UNH-129**	quality	++	++	++	++	++	++	++	++	++				--	++	-
	# all.	7	2	2	4	6	6	1	5	2+N	N	N	N	2	1	2
	all.range	189-215	199-201	199-203	187-201	185-209	183-209	209	175-217	183-209				193-201	203	237-253

Table S1 (continuation)

**Species**		***Oreochromis***							***Sarotherodon***		***Tilapia***			***Hemichromis***	***Chromidotilapia***	***Haplochromis***
**Loci**		***niloticus***	***aureus***	***mossambicus***	***shiranus***	***saka***	***squamipinnis***	***macrochir***	***melanotheron***	***galilaeus***	***zillii***	***dageti***	***guineensis***	***bimaculatus***	***guntheri***	***sp. rockkribensis***
**UNH-130**	quality	++	++	-	++	+ (echo)	+ (echo)	++	++	++	++	++	++			++
	# all.	7	2	2+N	5	4+N	4	2	1+N	5	1+N	2	2	N	N	2
	all.range	178-236	183-189	179-236	179-232	174-216	174-200	176-186	176	184-242	183	183	181-187			180-230
UNH-131	quality	+	--	-	-	-	-	-	+	-	--	--	--	--	--	--
	# all.	4	N	N	3	1	2	2	2	3	N	N	N	N	N	N
	all.range															
**UNH-132**	quality	++	++	++	++	++	++	++	++	++						
	# all.	2	3	3	5	1	1	2	1+N	4+N	N	N	N	N	N	N
	all.range	118-122	124-134	122-132	114-130	107	107	107-111	100	110-122						
**UNH-135**	quality	+ (echo)	+ (echo)	+ (echo)	+ (echo)	+ (echo)	+ (echo)	+ (echo)	+ (echo)	+ (echo)	+	+	+			
	# all.	6	2	4	3	9	8	3	5	7	3	3	5	N	N	N
	all.range	126-274	162-168	124-162	126-168	124-159	129-276	128-174	126-211	160-286	232-284	266-274	246-280			
**UNH-138**	quality	++	++	++	++	++	++	++	++	++	++	++	++			+
	# all.	7	1	1	8	8	8	3	2	9	5	3+N	5	N	N	1+N
	all.range	160-198	180	150	168-218	178-238	172-250	178-186	154-164	144-232	148-230	144-154	154-165			158
**UNH-142**	quality	++	++	++	++	++	++	++	++	++	++	++	++	+		+
	# all.	3	1	4	5	8	5+N	1	3	4	1	1	7	1	N	2
	all.range	156-160	156	146-172	152-168	156-192	152-178	172	146-160	142-154	142	142	142-186	142		170-176
**UNH-146**	quality	++	++	++	++	++	++	++	++	++	++	++	++		+	
	# all.	3	1	1+N	1	6	4+N	2	3+N	4	2	1	3	N	1	N
	all.range	123-131	125	123	125	129-145	129-137	133-149	121-133	111-147	111-115	111	111-117		113	
**UNH-149**	quality	++	++	++	++	++	++	++	++	+ (echo)	++	--	++	+	++	
	# all.	4	3	1	8	6	5	3	3	10	2	7	7	5	3	N
	all.range	145-161	149-193	147	159-200	145-157	145-159	183-225	163-177	143-203	161-167	153-221	149-183	161-177	159-163	
**UNH-154**	quality	++	+ (echo)	++	++	++	++	++	++	++	++	++	++	+	++	
	# all.	8	1+N	1	9	9	9	2	6	7	2+N	2	3	2	1	N
	all.range	100-174	124	128	108-164	108-166	112-176	138-158	102-120	100-136	118-158	104-108	98-104	98-112	114	
**UNH-159**	quality	++	++	++	++	+	+	+	++	++	++	+	++	-	++	-
	# all.	5+N	2	3	4+N	7	8	2	3	5	2	3	5	1	1	1+N
	all.range	229-267	239-243	229-257	221-247	227-267	227-249	255-259	217-237	229-241	208-218	231-234	210-245	209	205	211
**UNH-162**	quality	++	++	++	++	++	++	++	++	++	++	+	+	--	+	
	# all.	6	2	2	8	9	9	1	2	7	4	4	6	2	2	N
	all.range	200-238	198-200	208-230	186-216	206-252	204-242	194	181-210	191-230	212-244	125-229	205-240	209-217	202-204	

Table S1 (continuation bis)

Species		*Oreochromis*							*Sarotherodon*		*Tilapia*			*Hemichromis*	*Chromidotilapia*	*Haplochromis*
Loci		*niloticus*	*aureus*	*mossambicus*	*shiranus*	*saka*	*squamipinnis*	*macrochir*	*melanotheron*	*galilaeus*	*zillii*	*dageti*	*guineensis*	*bimaculatus*	*guntheri*	*sp. rockkribensis*
**UNH-169**	quality	++	++	++	+	+	+	++	++	++	++	++	++	++	++	++
	# all.	8	3	3	5	9	9	1	4+N	5	1	1+N	2	1	1	2
	all.range	124-166	132-192	148-168	152-184	144-220	144-240	140	136-168	136-156	136	136	134-136	144	172	134-144
**UNH-173**	quality	++	++	++		+	+	++	++	-	++	-	-			
	# all.	2	2	1	N	3+N	5+N	1	1+N	4	1	3+N	2	N	N	N
	all.range	172-188	174-180	172		178-188	176-188	160	154	136-172	124	136-142	124-140			
**UNH-174**	quality	++	++	++	++	++	++	++	++	++	+ (echo)	+ (echo)	++			++
	# all.	4	1	3	2+N	1	1	1	1	5	3	1	3	N	N	1
	all.range	165-187	153	151-181	146-151	149	149	155	151	149-161	157-161	159	165-181			154
**UNH-189**	quality	++	++	++	++	+	++	++	++	-	++	++	-		-	
	# all.	3	3	2	8	7	6	2+N	3	7	2	6	6	N	2	N
	all.range	161-190	152-184	146-184	142-171	150-198	154-208	146-156	155-161	149-198	140-144	149-203	135-173		135-138	
**UNH-190**	quality	++	++	++	++	++	++	++	++	++	++		+ or nul		++	-
	# all.	2	1	1	8	3	2+N	2	1	2+N	3	N	1+N	N	1	1
	all.range	168-177	170	141	143-192	141-145	141-143	141-149	145	147-159	186-198		202		133	198
UNH-193	quality	++	++	++	++	-- (or nul)	-- (or nul)	-- (or nul)	++	-- (or nul)	++	++	--	--	-- (or nul)	--
	# all.	3	2	2	2	N	N	N	3	N	1	1	N	N	N	N
	all.range															
**UNH-197**	quality	+ (echo)	+ (echo)	+ (echo)	++	+ (echo)	+ (echo)	++	+ (echo)	-	+	-	-		-	-
	# all.	6	2	2	6	7	9	1	5	9	4	5+N	6+N	N	3+N	2
	all.range	156-206	174-186	170-184	200-218	164-198	154-210	190	202-228	188-222	180-198	191-218	178-199		164-194	214-220
**UNH-207**	quality	+ (echo)	++	++	++	++	++	++	+ (echo)	+ (echo)	++	++	++		++	+
	# all.	3	1	2+N	2	5	8	1	4	7	2	3	5	N	1	2
	all.range	140-158	148	110-116	116-148	116-164	112-166	116	118-154	114-166	128-134	112-174	114-144		90	184-198
**UNH-211**	quality	++	++	++	++	++	++	++	++	++	++	-	-			
	# all.	6	2	2	7	9	8	2	8	5+N	2	1	4	N	N	N
	all.range	128-162	134-162	144-160	144-180	142-176	124-184	128-158	154-194	136-170	82-84	82	84-194			
**UNH-216**	quality	++	++	++	++	++	++	++	++	++	++	+	++	--		++
	# all.	2	1	2	9	7+N	8	1	4	7	2	7	6	4+N	N	2
	all.range	126-138	128	160-174	152-178	160-186	146-168	176	136-170	136-190	160-174	138-168	150-174	168-212		160-190
**amplification rate**		97%	88%	88%	88%	88%	88%	91%	97%	75%	75%	63%	63%	19%	47%	38%
**P(0.95)**		97%	57%	71%	93%	89%	89%	55%	77%	96%	42%	60%	80%	50%	20%	50%
**mean.all./Locus**		4.3	1.8	2.1	5.2	5.9	5.8	1.7	2.8	4	1.5	2.4	3.6	2.3	1.4	1.6

Table S2 Cross-species amplification results per taxonomic groups (genus, tribe & total of species) with the number of alleles, calculated i) per locus and ii) over all loci (with amplification and polymorphism rates, mean alleles number per locus & mean alleles number per locus and species); expected null alleles (N) are reported.

**Taxonomic groups Loci**	***Oreochromis***	***Sarotherodon***	***Tilapia***	**Tilapiines**	**non-Tilapiines**	**Total**
**UNH-008**	16+N	6	1	20+N	4	22+N
**UNH-102**	16+N	5	0+N	17+N	4+N	19+N
**UNH-103**	27	8	0+N	31+N	0+N	31+N
**UNH-106**	20	6+N	7+N	28+N	8+N	30+N
**UNH-115**	12	4	3	14	2+N	15+N
**UNH-117**	2+N	3	2	6+N	3	9+N
**UNH-123**	20+N	6	2+N	23+N	3	26+N
**UNH-124**	6+N	5	2+N	12+N	0+N	12+N
**UNH-125**	12+N	4+N	5	16+N	1+N	17+N
**UNH-129**	16	6	0+N	19+N	5	21+N
**UNH-130**	19+N	5+N	4+N	25+N	2+N	26+N
**UNH-132**	12	5+N	0+N	14+N	0+N	14+N
**UNH-135**	23	7	8	33	0+N	33+N
**UNH-138**	25	8	11+N	38+N	1+N	38+N
**UNH-142**	15+N	4	7	18+N	3+N	18+N
**UNH-146**	9+N	5	4	16+N	1+N	16+N
**UNH-149**	19	9	14	31	6+N	31+N
**UNH-154**	28+N	8	6	32+N	3+N	33+N
**UNH-159**	17+N	7	9	26+N	3+N	29+N
**UNH-162**	22	7	10	34	4+N	35+N
**UNH-169**	28	7+N	2+N	31+N	3	31+N
**UNH-173**	10+N	3+N	5+N	17+N	0+N	17+N
**UNH-174**	10+N	3	4	11+N	1+N	12+N
**UNH-189**	21+N	8	12	36+N	2+N	37+N
**UNH-190**	14+N	3	4+N	18+N	2+N	19+N
**UNH-197**	25	11	13+N	36+N	5+N	38+N
**UNH-207**	13+N	8	9	23+N	3+N	26+N
**UNH-211**	24	11	5	31	0+N	31+N
**UNH-216**	18+N	7	11	25+N	6+N	29+N
**amplification rate**	88%	86%	67%	82%	34%	72%
**P (0.95)**	76%	85%	59%	74%	36%	70%
**mean.all.nb./Locus**	17.8	6.4	6	24.3	3.4	25.7
**mean.all.nb./Loc./sp.**	3.7	3.3	2.4	3.7	1.6	3.2

Table S3 Number and percentage of shared alleles between species by taxonomic groups (genus, tribe & total of species), calculated per locus and in average over all loci (calcul of percentage excludes null alleles).

Taxonomic groups Locus		*Oreochromis*	*Sarotherodon*	*Tilapia*	Tilapiines	non-Tilapiines	Total
**UNH-008**	# all.	6	1	1	3	0	2
	% all.	37.5%	16.7%	100%	15%	0%	9.10%
**UNH-102**	# all.	9	1	N	4+N	0	2+N
	% all.	56.3%	20%	-	23.50%	0%	10.50%
**UNH-103**	# all.	9	0	N	4	N	N
	% all.	33.3%	0%	-	12.9%	-	0%
**UNH-106**	# all.	5	1	0	5	0	6+N
	% all.	25%	16.70%	0%	17.90%	0%	20%
**UNH-115**	# all.	7	1	1	3	0	1
	% all.	58.3%	25%	33.3%	21.4%	0%	6.7%
**UNH-117**	# all.	1	0	1	1	0	0
	% all.	50%	0%	50%	16.70%	0%	0%
**UNH-123**	# all.	5+N	0	1	4+N	0	0
	% all.	25%	0%	50%	17.4%	0%	0%
**UNH-124**	# all.	3+N	1	N	1+N	N	N
	% all.	50%	20%	0%	8.30%	-	0%
**UNH-125**	# all.	N	0	1	4+N	N	N
	% all.	0%	0%	20%	25%	0%	0%
**UNH-129**	# all.	7	0	N	3+N	0	3+N
	% all.	43.80%	0%	-	15.80%	0%	14.30%
**UNH-130**	# all.	6+N	0	1	4+N	N	1+N
	% all.	31.6%	0%	25%	16%	0%	3.8%
**UNH-132**	# all.	3	0	N	2+N	N	N
	% all.	25%	0%	-	14.30%	-	0%
**UNH-135**	# all.	9	0	1	4	N	0
	% all.	39.1%	0%	12.5%	12.1%	-	0%
**UNH-138**	# all.	9	0	1	1	N	1
	% all.	36%	0%	9.1%	2.6%	0%	2.6%
**UNH-142**	# all.	9	1	1	8	0	3+N
	% all.	60%	25%	14.3%	44.4%	0%	16.7%
**UNH-146**	# all.	6+N	1	1	2	N	1+N
	% all.	66.7%	20%	25%	12.5%	0%	6.3%
**UNH-149**	# all.	7	0	2	7	2	6
	% all.	36.8%	0%	14.3%	22.6%	33.3%	19.4%
**UNH-154**	# all.	8	2	1	7	0	2+N
	% all.	28.6%	25%	16.70%	21.90%	0%	6.10%
**UNH-159**	# all.	8	1	0	6	0	N
	% all.	47.1%	14.3%	0%	23.1%	0%	0%
**UNH-162**	# all.	10	0	2	4	0	3
	% all.	45.5%	0%	20%	11.80%	0%	8.60%
**UNH-169**	# all.	9	2	1	6	1	3
	% all.	32.1%	28.6%	50%	19.4%	33.3%	9.7%
**UNH-173**	# all.	3	0	1	1	N	N
	% all.	30%	0%	20%	5.90%	-	0%
**UNH-174**	# all.	3	1	1	6	N	N
	% all.	30%	33.3%	25%	54.5%	0%	0%
**UNH-189**	# all.	6	0	1	5	N	1+N
	% all.	28.60%	0%	8.30%	13.90%	0%	2.70%
**UNH-190**	# all.	2	0	N	3	0	1+N
	% all.	14.3%	0%	0%	16.7%	0%	5.3%
**UNH-197**	# all.	6	0	N	12	N	3+N
	% all.	24%	0%	0%	33.30%	0%	7.90%
**UNH-207**	# all.	4	0	1	7	0	N
	% all.	30.8%	0%	11.1%	30.4%	0%	0%
**UNH-211**	# all.	9	1	2	9	N	0
	% all.	37.50%	9.10%	40%	29%	-	0%
**UNH-216**	# all.	9	1	3	9	N	1+N
	% all.	50%	14.30%	27.30%	36%	0%	3.40%
**Average per locus**	# all.	6.14	0.52	0.86	4.66	0.1	1.38
	% all.	37%	9.2%	19.7%	20.5%	2.3%	5.3%
